# Zinc level assessment in patients having Viral Cirrhosis

**DOI:** 10.12669/pjms.342.14457

**Published:** 2018

**Authors:** Lubna Kamani, Hafeezullah Shaikh

**Affiliations:** 1 Dr. Lubna Kamani, MBBS, MRCP (UK), FRCP, FCPS. Associate Professor, Department of Gastroenterology, Liaquat National Hospital, Karachi, Pakistan; 2 Dr. Hafeezullah Shaikh, MBBS, FCPS, Consultant Gastroenterologist, Department of Gastroenterology, Liaquat National Hospital, Karachi, Pakistan

**Keywords:** Cirrhosis, Hepatitis, Hepatic encephalopathy, Zinc level

## Abstract

**Objective:**

Zinc is a vital trace element and its deficiency in cirrhosis might potentiate the development of hepatic encephalopathy. The objective of this study was to assess the zinc levels in serum of patients having viral cirrhosis and compare it with normal healthy controls.

**Methods:**

This study was conducted in Department of Gastroenterology, Liaquat National hospital and medical college, Karachi, Pakistan; from January 2014 to December 2014. Total of 45 patients with the mean age of 52.44±8.7 years were included. The three groups of patients were made including Child Pugh Class Score B (Group-1), Child Pugh Class C (Group-2) and healthy controls (Group-3) having 15 patients in each group. Zinc levels in serum were evaluated by the help of atomic absorption spectrometry (Normal range50-150 µg/dl).

**Results:**

Total of 45 subjects was enrolled in this study. Overall prevalence of zinc deficiency was noted in 13(28.9%) patients. Mean value of zinc levels in group 1,2 and 3 were 68.09±20.85, 50.69±15.86 and 92.91±17.18µg/dL respectively. Highly statistical difference was observed in the mean zinc level between three groups p=0.0001. An inverse correlation was observed between Child Pugh Score and the zinc level in serum r=-0.498.

**Conclusion:**

Patients suffering from advanced cirrhosis appeared to have lower serum zinc levels. In patients suffering from viral cirrhosis having hepatic encephalopathy, zinc supplementation might improve clinical outcome.

## INTRODUCTION

The usual cause of mortality in hospital globally is cirrhosis of Liver as a consequence of hepatocellular damage which results in fibrosis and nodular regeneration throughout the liver.^1^ Cirrhosis is very communal disease in Pakistan, frequently initiated by hepatitis C virus followed by hepatitis B virus or a combination of both.^2^,^3^ There has been a decline in zinc concentrations in serum in patients suffering from liver cirrhosis resulting in portosystemic shunting which further augment the deficiency.^4^ Zinc functions as antioxidant and is an important trace element that enhances the effectiveness of urea cycle as well.^5^ The commonest site for the absorption of Zinc is jejunum, followed by the ileum and duodenum. Zinc is one of the most vital minerals of the body as it is required for the activity of greater than 100 enzymes. Zinc also participates as an important substance in growth and differentiation of cells. Vitamins and minerals are needed in trace amounts and assist as parts of enzymes or coenzymes for the essential metabolic reactions in the body. The patients of decompensated chronic liver disease with hepatic encephalopathy suffer from the insufficiency of zinc. Therefore patient suffering from decompensated cirrhosis shows zinc deficiency as liver is the structure with maximum zinc utilization.

The most common complications as consequence of live cirrhosis are ascites, portal hypertension, hepatic encephalopathy and varices. However, the possibility of zinc insufficiency should be taken into consideration which might be the concealed accelerating reason for the establishment of hepatic encephalopathy in later stages of the damage^6^ and thus it should be ruled out. Deficiency of zinc might be the reason for many systematic dysfunctions, including neurologic (may result in hepatic encephalopathy), hepatobiliary, reproductive, immune and skeletal systems. Conversely, the zinc supplementation considerably recovers neurologic signs in cirrhotic patients with refractory hepatic encephalopathy.^7^,^8^

One of the Study done by Gusau KA et al. discovered a significant decrease in the serum zinc levels of African cases when related to the controls.^9^ It is proposed that the deficiency of zinc in serum of liver cirrhosis patients, influence and potentiate the development of hepatic encephalopathy.^8^ The frequency of zinc insufficiency has been stated in alcoholic cirrhosis but there is a lack of these values in viral cirrhosis. This study was designed to assess and evaluate the zinc levels in serum of patients with viral cirrhosis.

## METHODS

This was an observational study conducted at Department of Gastroenterology, Liaquat National Institute for Postgraduate Medical Studies and Health Sciences, Karachi, Pakistan from January 2014 to December 2014. Overall 45 consecutive patients, aged range between 25-75 years of both gender (15) patients each in child class B and C viral cirrhosis and healthy controls were enrolled. Patients with acute infection, on zinc supplementation, chronic diarrhea and hemolytic disorder were excluded.

This study was conducted on ethical standards of the institute, fully informed and written permission was attained from the participants prior to the conduction of this study and there was no conflict of interest. Thorough history was taken including medication history. Systemic and local examination was done with particular emphasis on abdomen, to assess ascites, hepatosplenomegaly, jaundice, further complains and findings of chronic liver disease. Presence of variceal upper GI bleeding, ascites, hepatocellular carcinoma or hepatic encephalopathy was considered as decompensated chronic liver disease. Ultrasound was done to rule out hepatocellular carcinoma and ascites.

A 3ml blood sample was obtained for the zinc assay with care and under vigilance of doctor following standard protocols of sterilization using a syringe, and transferred to zinc-free test tubes. The samples were drawn in the day time and sent to institutional laboratory. Zinc levels in serum were measured using atomic absorption spectrometry by a laboratory technician unaware of the study. Zinc level between 50-150µg/dlwas considered as normal.

### Statistics analysis

Data was inserted and analyzed on SPSS version 17. For quantitative variables Mean±SD were considered and frequency and percentages were calculated for quantitative variables. Zinc level was compared between child class B, C and healthy controls by applying one way ANOVA. The correlation coefficient between the serum zinc levels and the Child-Pugh scores was determined and a p value ≤ 0.05 was considered as significant.

## RESULTS

Total of 45 subjects were enrolled in the study. Patients were divided into three groups. Child Pugh Class Score B (Group-1), Child Pugh Class C (Group-2) and healthy controls (Group-3) having 15 patients in each group. The base line characteristics of all patients are shown in Table-I. The healthy controls were significantly younger as compared to cases 42.47±10.96 years, (p 0.002). In the etiology of liver cirrhosis, hepatitis C cases were more as compared to hepatitis B but had no significant difference in class B and C Child Pugh groups. All patients in Group-2 and 3 had decompensated liver disease and there were no difference in hepatic encephalopathy grades. Child Pugh Class C had ascites in higher number of patients 14 (93.3%) when compared with Child Pugh Class B, 6(40%), which had statistical significance p= 0.002. Overall prevalence of zinc deficiency was noted in 13(28.9%) patients suffering from cirrhosis, 4 (26.7%) patients in Group 1 and 9 (60%) patients in Group-2. Mean value of zinc levels in Group 1,2 and 3 were 68.09±20.85, 50.69±15.86 and 92.91±17.18 µg/dL respectively. On applying one way ANOVA, highly statistical difference was observed in the mean zinc level between three groups p=0.0001. Statistically significant difference in zinc level deficiency was noted in Group-2 and 3, p=0.001. An inverse correlation was observed between Child Pugh Score and the serum zinc level r=-0.498. Overall prevalence of zinc deficiency was noted in 13(28.9%) patients suffering from cirrhosis, 4 (26.7%) patients in Group1 and 9 (60%) patients in Group-2.

**Table-I:** Demographic characteristics and comparison of groups (n=45)

Characteristics	Child Pugh Class B n=15 (Group-1)	ChildPugh Class C n=15 (Group-2)	Healthycontrols n=15 (Group-3)	P-value
Age in years (Mean ± SD)	58.93±9.99	55.93±16.36	42.47±10.96	0.002*
Male (%)	6(40)	5(33.33)	6(40)	0.910
HBV	3(20)	5(33.3)	-	0.682
HCV	12(80)	10(66.7)	-	0.682
Decompensated	15(100)	15(100)	-	-
*Encephalopathy grades*				
Grade I	1(6.7)	0(0)	-	0.473
Grade II	4(26.7)	3(20)	-	0.473
Grade III	1(6.7)	2(13.3)	-	0.473
Grade IV	0(0)	2(13.3)	-	0.473
Ascites	6(40)	14(93.3)	-	0.002*
HCC	1(6.7)	4(26.7)	-	0.330
UGIB	3(20)	3(20)	-	1.000
Score	8.73±0.458	11.27±1.534	-	0.000*

HBV: hepatitis B virus, HCV: hepatitis C virus, HCC: Hepatocellular carcinoma,

UGIB: upper gastro-intestinal bleeding,

* Statistically significant, number (%)

## DISCUSSION

Zinc is among the most abundant intracellular trace element found in the body performing vital functions in various biochemical reactions. Zinc insufficiency disrupts numerous organ and systems of the body including gastrointestinal system that further reduces the functions of liver. The maximum uptake of zinc after assimilation occurs in liver, consequently chronic liver disease leads to the deficiency of zinc.

The reduction in zinc levels can decline the immune function, cognition, and growth of the body.^10^-^13^ Moreover, it can occur with liver cirrhosis, leading to unusual ammonia levels and various other substances due to improper metabolism of protein, and the commencement of hepatic encephalopathy. International studies on zinc supplementation therapy in patients having cirrhosis of liver have revealed to improve hyperammonemia and hepatic encephalopathy due to enhancement in protein metabolism.^14^,^15^

The function of zinc in nourishment and development of children suffering chronically from liver disease is imprecisely demarcated. In a study on 90 children having liver disease chronically, 30 patients had age of 4.5 years and Child Pugh score was 5.83, 30 with decompensated chronic liver disease having the age of 1.39 years as mean and Child Pugh score of 9.53, and 30 healthy children having the mean age of 4.6 years, (16 boys), it was found that Zinc levels of patients with compensated liver disease were prominently decreased as compared with the healthy controls; 68.07±31.55vs 89.9 ±25.9microg/dL, P = <0.001), but considerably raised associated to the patients with decompensated liver disease (48.8 [26.8] microg/dL). Furthermore, it was found that an inverse correlation existed between the Child-Pugh score and zinc levels (r = -0.460).^16^

The presence of control group in our study confirms the association of low serum zinc level and chronic liver disease. Zinc deficiency was observed in both Child Pugh class B and C but higher was noted in class C as compared to class B in our study. The results of our study were in cognition with the results of a study done by Poo et al.^17^ one of the Revision by Gusau KA et al. discovered a remarkable decline in serum zinc levels in African cirrhotic patients when related with the controls.^9^ The several reasons that are responsible for this reduction could be due to malabsorption of zinc and post bacterial advancement from compromised small bowel motility.^18^ Moreover, as all the patients were of decompensated liver disease, the phenomena of low serum zinc level could be due to poor intake because of dysgeusia, repeated infections, and reduced appetite that is caused by massive ascites, which was present in majority of our subjects.^19^

Grungrieff K et al. concluded that zinc supplementation in in daily dosage limits is quite safer and may improve liver cirrhosis.^20^ Gur G et al. in his study found that the mean liver zinc concentrations were 3.83 ± 1.86, 1.86 ±0.92, 1.14 ± 0.68 and 3.74 ± 1.81mumol/g among control group, active chronic hepatitis, cirrhosis and persistent chronic hepatitis groups respectively.^5^

Thus, many studies have established that higher the severity of liver disease, lower would be the serum zinc level.^5^,^9^,^16^-^19^ In one study Mosua N et al. found improvement of minimal hepatic encephalopathy in cirrhosis after zinc supplementation.^21^ Therefore, it can be proposed that zinc supplementation may reduce hepatic encephalopathy by improving the effectiveness of the urea cycle.

The strength of our study is that we have sampled the patients with different grades of the chronic liver disease. However, the study may have some observational bias. Considering the results of zinc levels in our study and to what extend they might be consistent with the other types of liver diseases would be enlightening and valuable to classify more facts about the patients of liver cirrhosis.

## CONCLUSION

The zinc levels in patients with cirrhosis were considerably reduced and were found to be inversely related to the severity of the liver disease. Therefore, the Zinc supplements might reduce the clinical worsening of the cirrhotic patients with complications. However, more research based evidences are required in Pakistan to elaborate the role of zinc in cirrhotics.
